# Adequate sensing of ventricular fibrillation?

**DOI:** 10.1007/s12471-017-0996-x

**Published:** 2017-04-26

**Authors:** A. W. G. J. Oomen, B. M. van Gelder, F. A. L. E. Bracke

**Affiliations:** Catharina Hospital, Eindhoven, The Netherlands

## Answer

**Fig. 1:**
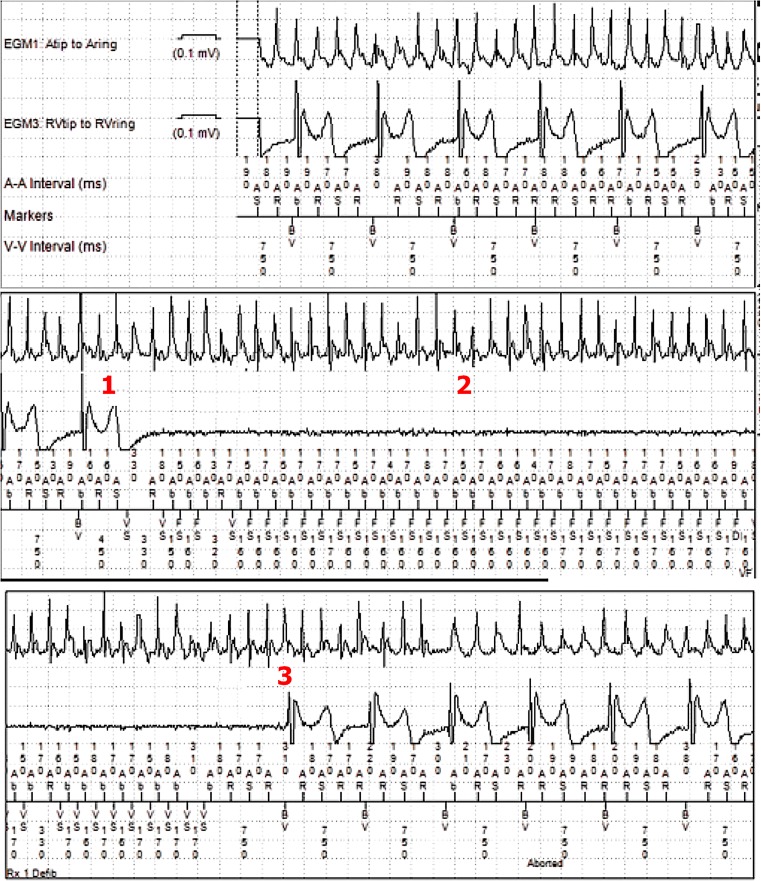
After atrial fibrillation with biventricular pacing (upper panel), there is a sudden change initiated by ventricular oversensing *(1)*. Subsequently, atrial fibrillation is sensed in the ventricular channel *(2)*; note the synchrony between atrial and ventricular sense markers in the lower channel. There is a spontaneous abortion of atrial oversensing *(3)* and restoration of biventricular pacing

The first part of the recording (Fig. 1) shows continuous atrial fibrillation (AF) with biventricular pacing. After a single T‑wave is oversensed (1), the device automatically adjusts the sensitivity based on the amplitude of the preceding sense event [1]. This more sensitive setting leads to detection of AF in the ventricular channel, interpreted as ventricular fibrillation (VF) and abortion of ventricular pacing (2). After approximately 9 s, AF oversensing has stopped spontaneously, biventricular pacing is resumed and VF therapy is aborted (3).

**Fig. 2:**
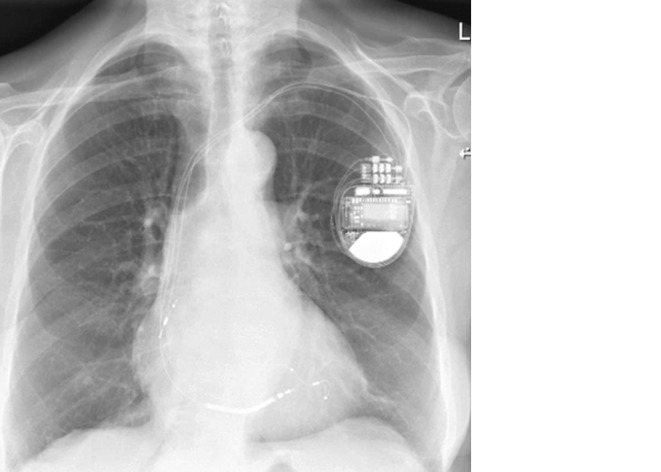
Posterior-anterior chest X‑ray, showing the RA lead, a quadripolar LV lead and RV shock lead. Note the position of the proximal segment of the shock lead, which is most likely located in the lower right atrium at the level of the coronary sinus

It is important to realise that, in contrast with the RV tip to ring configuration default depicted on the marker channel, this biventricular implantable cardioverter-defibrillator was programmed RV tip to RV coil. AF sensing in the RV lead is proven by the synchronous atrial and ventricular sensing in Fig. 1 (question) and Fig. 1 (answer). Near-field sensing of AF with the ventricular lead is explained by the use of the coil as anodal electrode for pacing and sensing with the proximal part located in the lower right atrium (Fig. 2).

In this case, the key problems were the position of the RV lead and the coil being used as a proximal sensing electrode. Once the device was reprogrammed to tip to ring, the problem was solved. Patient had a good clinical response to biventricular pacing and is doing well.

Although this is a rare phenomenon, the lesson to be learned is that, when we change the sensing vector to an integrated bipolar setting, we must make sure that the coil is well within the right ventricle.
